# Design and Reliability of an Observational Framework to Evaluate the Individual Offensive Behavior in Youth Soccer—The INDISOC Tool

**DOI:** 10.3390/children9091311

**Published:** 2022-08-29

**Authors:** Joaquín González-Rodenas, Iván Villa, Andrés Tudela-Desantes, Rodrigo Aranda-Malavés, Rafael Aranda

**Affiliations:** 1 Centre for Sport Studies, Rey Juan Carlos University, 28942 Madrid, Spain; 2 Department of Physical Education and Sports, University of Valencia, 46010 Valencia, Spain

**Keywords:** observational methodology, match analysis, football, youth development, technical demands

## Abstract

Despite the great development of match analysis in professional soccer during the last decade, very few studies have assessed the individual technical and tactical behaviors of youth soccer players. The purpose of this paper was to design and assess the reliability of an observational instrument to evaluate the INDIvidual offensive behavior in competitive 7 and 11-a-side SOCcer (INDISOC). A total of eight experts in soccer training and analysis were included in the design of the tool by means of meetings and exploratory observations. This process involved design and re-design steps of the INDISOC tool to its final format which includes twelve dimensions related to the spatial, technical, and tactical constraints of individual behavior in soccer. The unit of analysis was the individual ball possession (IBP), described as the time that starts when a player can perform an action with the ball, and which ends when the IBP for another player begins. In the INDISOC tool, the IBP is analyzed taking into account three temporal moments: (1) receiving the ball, (2) processing the ball, and (3) culminating the individual action. Inter-observer and intra-observer analyses were performed and the kappa (K) coefficients were calculated to test the instrument reliability. The K values showed optimal inter (7-a-side: 0.73–0.95; 11-a-side: 0.76–0.98) and intra-observer (7-a-side: 0.84–1;11-a-side: 0.79–1) reliability levels. These results support the notion that the INDISOC observational tool could be a suitable instrument for analyzing the individual offensive behavior in competitive youth (7-a-side), junior and senior (11-a-side) soccer.

## 1. Introduction

The complex nature of the game of soccer (association football), considered as a dynamic and interactive phenomenon, makes it complicated to objectify its evaluation [1]. The recent technological innovations in the field of match analysis are leading to an increasing volume of data in professional soccer [2,3]. This fact is also contributing to the publication of a higher number of research papers about teams’ performance during elite soccer competitions [4,5]. However, despite the great development of match analysis in the last years, there is limited research on the individual dimensions of performance in players, and especially in children [6,7]. Particularly, very few studies have assessed the individual technical and tactical performance of youth soccer players both in training and competition [8,9].

From a pedagogical perspective, evaluating the individual tactical performance may be crucial to design an appropriate learning process in youth ages. In this sense, Barreira, Casal, Losada, and Maneiro [10] suggested that the assessment of technical skills needs to be further developed in the natural context of soccer (i.e., small-sided games), by creating observational tools that allow to capture data during matches or other contextualized games. In this sense, one of the most important performance factors that discriminate between high-skilled and less-skilled soccer players is the ability to make optimal decisions during diverse match situations [11]. This ability allows players to solve different tactical situations with effectiveness under spatial and time constraints. For this reason, a key question for researchers in soccer is how to design observational tools to assess the tactical ability and talent of youth soccer players.

In this regard, systematic observation is considered a suitable methodology for analyzing tactical behavior [12] because it permits the inclusion of categorical data from the qualitative evaluation of different dimensions of match performance and may improve the ability to describe soccer match play actions [13,14]. From this methodology, a variety of observational tools have been created in the last decades to assess the players behaviors in soccer [15], such as the “Performance Assessment in Team Sports” (TSAP) [16], “Game Performance Assessment Instrument (GPAI) [17], “Procedural Tactical Knowledge Test (KORA) [18], “System of Tactical Assessment in soccer (FUT-SAT) [19], “Game performance evaluation tool” (GPET) [20], the “Instrument for Measurement of Learning and Performance in Football” (IMLPFoot) [21], the “observational tool for technical and tactical actions in the offensive phase in soccer [22], and the “Football Competence Observation System (FOCOS) [23].

These tools have provided interesting frameworks to analyze individual tactical behavior in soccer in different contexts. For instance, the GPAI, GPET, and KORA, designed for invasion sports, focus on evaluating the decision-making process of youth/beginner players (6–14 years old), based on the implementation of general principles of play related to the ball possession/progression and the numerical superiority or spatial advantage during small sided games. The IMLPFoot is a soccer-specific tool that focuses on the evaluation of the decision-making, technical execution, and results of the main offensive and defensive actions made by players during small sided games. Otherwise, The FUT-SAT instrument focuses on the tactical behavior, tactical performance, and decision making of players during the actual match play based on ten core tactical principles. However, this tool does not analyze the specific technical or tactical actions that the players display. The FOCOS tool analyzes the roles, the own actions of acquired subroles, and the principles adopted by the players in a small-sided game format (Gk + 4v4+ Gk), offering a complete analysis of the behaviors that the player can develop during their performance. Finally, the observational tool created by Ortega-Toro et al. [22] provides a technical–tactical analysis of the players during the start, development, and end of the individual ball possession. This interesting tool included the evaluation of dimensions related to the type of technical action, numerical situation in relation to the opposing team, and the offensive support of the player with the ball.

In general, the creation of these tools has helped researchers and coaches to perform studies about the technical and tactical skills in youth soccer players, as well as to explore the effect of different training tasks on the player’s actions. For instance, Castelao et al. [24] used the FUTSAT tool and observed that smaller training formats such as 3v3 are more likely to emphasize tactical principles such as delay and penetration, whereas larger formats (e.g., 5v5) can increase the utilization of principles such as defensive unity and balance. Another example is the study of Praxedes et al. [25], which used the GPET to evaluate the positive improvements in passing and dribbling of youth soccer players after a training intervention based on modified soccer games in youth players.

However, despite the variety and quality of the existing observational tools, it is relevant to mention that their design is primarily oriented to the analysis of tactical skills occurring in modified versions of soccer, such as small sided games, what may limit their application to analyze players in the real competition. This fact has certain advantages such as increasing player participation, reducing the complexity of the game, and controlling the tactical environment where the player is evaluated. Nevertheless, this aspect also has limitations such as not considering the effects of the contextual variables, as well as not analyzing the player under the real physical, spatial, and time constraints.

On the other hand, it is crucial to highlight that due to the complex and interdependent nature of game actions in soccer, the individual performance of soccer players depends on their interaction with teammates, opponents, and spatial–temporal constraints. Nevertheless, the mentioned tools are not designed to analyze the interaction of the observed players with their teammates to capture the connection between the individual player and the team. In addition, the majority of these instruments do not consider the effect of the interaction between the player’s actions and the opposing team’s behavior.

Therefore, the aim of this study was to design an observational tool to analyze the INDIvidual offensive behavior in SOCcer (INDISOC) in 7-a-side and 11-a-side competition, considering the interaction of the player with the collective offensive behavior and the opposing team spatial and organizational constraints. The main objective of this tool is to help coaches and researchers to evaluate the tactical ability especially of young and junior players, but it is also applicable to senior players.

## 2. Methods

### 2.1. Participants

A total of eight experts participated throughout the process of designing the INDISOC observational tool, who met at least three of the following four inclusion criteria: (1) graduate in Sport Sciences, (2) hold a Soccer Coach UEFA Pro License, (3) have more than 5 years of soccer coaching experience at the academy or senior level, (4) have a PhD in observational methodology in team sports.

### 2.2. Procedure

The research design followed four steps based on the methodology used in previous studies that validated observational tools in team sports [22,26,27,28]. In the first step, a review of the literature was conducted to establish an exhaustive list of previously published observational tools and technical behaviors analyzed in research studies (the major databases explored were SportDiscus^®^, PubMed, Web of Science, Google Scholar, and Dialnet). As for previous observational tools, it is important to highlight the most recent instruments for the individual analysis in soccer such as the FUT-SAT [19], GPET [20], S-SBMT [29], IMLPFoot [21], and FOCOS [23]. It is crucial to mention that other studies such as the one of Ortega-Toro et al. [22] developed the idea of considering three different moments within the IBP, which offered another perspective in the analysis of technical and tactical actions in soccer. In this manner, the player´s behavior could be evaluated according to several dimensions at the start, development, and the end of IBPs. Regarding the technical actions, previous studies usually analyzed ball technical actions such as “passes, touches per possession, passes towards the opponent goal, successful passes, shots, crosses, dribbles, clearances, aerial challenges, interceptions, losses of control, tackles, corners, free kicks, throw-ins, and rules breached” [30,31]. 

The second step consisted of designing a proposal for elaborating an observational tool for the analysis of individual tactical behavior in soccer. For this purpose, a group of four internal experts was created and group meetings were conducted to develop the tool construction. The group of experts set the objective to create a tool that was able not only to analyze the technical actions but also to evaluate the tactical context where the actions take place according to the collective behavior and opponent interaction. With this idea, the tool must include the analysis of individual, environmental, and task constraints, such as previous studies claimed [32,33]. Additionally, the group of experts decided to focus on the analysis of the attacker with the ball, discarding the analysis of defensive actions or offensive actions off the ball. The attacker with the ball includes the behavior of goalkeepers, although no specific dimensions are created for this field position as in the study of Jara et al. [28]. This decision, despite the limitations it entails, was made to design a very specific tool based on offensive actions with the ball.

In this way, practices of exploratory observation to identify potential dimensions and categories were carried out by the experts. Initially, the unit of analysis was exclusively the technical–tactical action developed by the player, including one only temporal moment. In this stage, a total of 22 possible dimensions were identified and explored. However, the re-design carried out throughout the exploratory observations reduced the dimensions to 16, discarding those dimensions less relevant and those who provided redundant information. Additionally, due to the complexity and temporal variability of the players’ tactical behaviors, different temporal moments were identified within the individual sequences. In this manner, the unit of analysis changed from the technical–tactical action to the “individual ball possession” [34], differentiating three temporal moments during the IBP (receiving the ball, processing the ball, culminating the action), which is similar to the observational tool created by Ortega-Toro et al. [22]. Once the main variables and categories were developed and no more appearance of new behaviors was detected during exploratory observation, a theoretical document including operational definitions and graphic representations was made.

In the third step, content validity was established through the consultation of other four external experts and Aikens´s V coefficient was calculated [35]. In this regard, group meetings with these experts were set to analyze the content of the theoretical framework of the INDISOC tool. During these meetings, the experts were asked about (a) the level of comprehension of the operational definitions of the dimensions from the observational tool; (b) the level of pertinence of these dimensions; (c) the need to include other dimensions in the observational tool; and (d) the overall evaluation of the observational tool. In this process, the dimensions of the INDISOC tool were reduced from 16 to 12, focusing on those dimensions more relevant and easier to analyze and interpret, according to the suggestions made by the external experts.

In the fourth step, the inter-observer and intra-observer reliability was tested for both 7-a-side and 11-a-side soccer. For this purpose, two observers were trained in the use of the INDISOC tool for four weeks. This training included theoretical and practical lessons, exploratory observations, and discussion between the observers and the internal experts. For 7-a-side soccer, the observer and the main researcher analyzed 163 IBPs corresponding to two matches of the U12 tournament LaLiga *Promises* 2021. For 11-a-side soccer, the other observer and the main researcher analyzed 103 IBPs performed by the Spanish player Pedri during the match Spain versus Sweden in the 2020 Eurocup. For the analysis of interpretative stability, the main researcher re-observed the IBPs four weeks later to check the intra-observer reliability.

Ethical approval was not required for this study because the tactical analyses of players were performed in matches that were recorded from TV broadcasters and the videos were public. In this regard, confidentiality was not an issue and authorization was not required from the observed players, so that the research consisted solely of naturalistic observation in a public TV broadcast of a routinary competition of soccer players in their teams [36,37] where no invasive, individual, or identifiable measures were performed.

### 2.3. Observational Instrument

The INDISOC instrument is a combination of field format and category systems [38]. The observation design is punctual, because the data collection takes place in one single session, idiographic, because it is focused on a study unit (the player), and multidimensional, because the player´s performance is based on various criteria [39].

**Figure 1:**
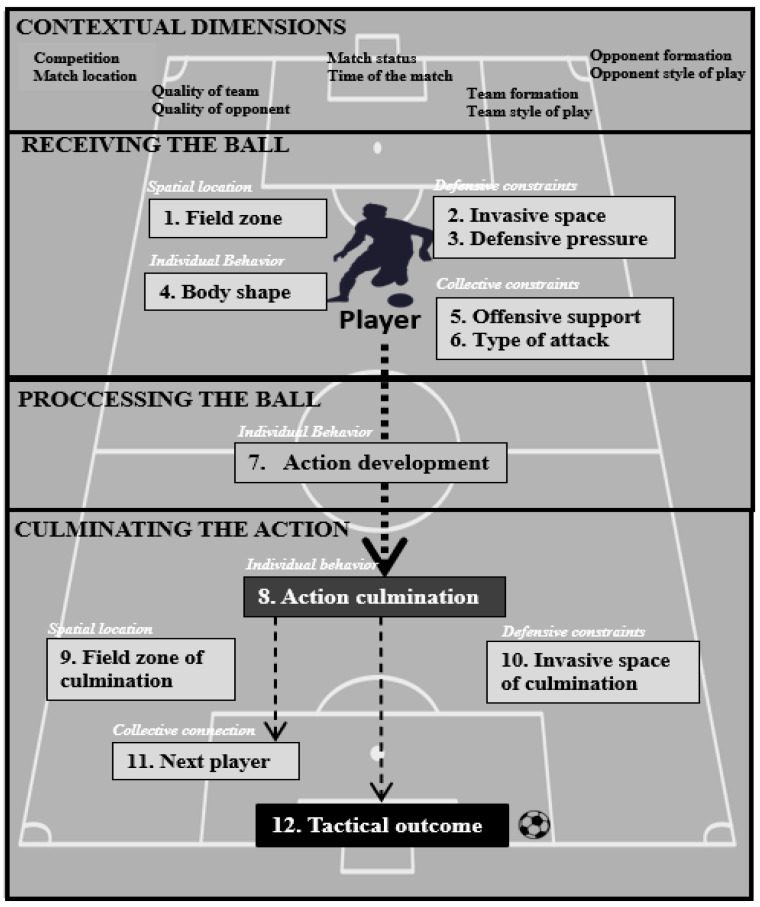
Scheme of the macro-criteria, temporal moments, and dimensions of the INDISOC tool.

The unit of analysis is the IBP, described by Link and Hoernig [34] as the time that starts when a player can perform an action with the ball (following an IBP of another player or a game interruption) and it ends when an IBP for another player begins. As is shown in Figure 1, the IBP is evaluated in three different moments: (1) receiving the ball: this period involves just the moment when the observed player comes into contact with the ball; (2) processing the ball: this period covers the time since the player comes into contact with the ball until he/she stops being in contact with it; and (3) culminating the action: this period covers the time since the player makes the last contact with the ball until a tactical outcome is achieved.

### 2.4. Receiving the Ball

**Figure 2:**
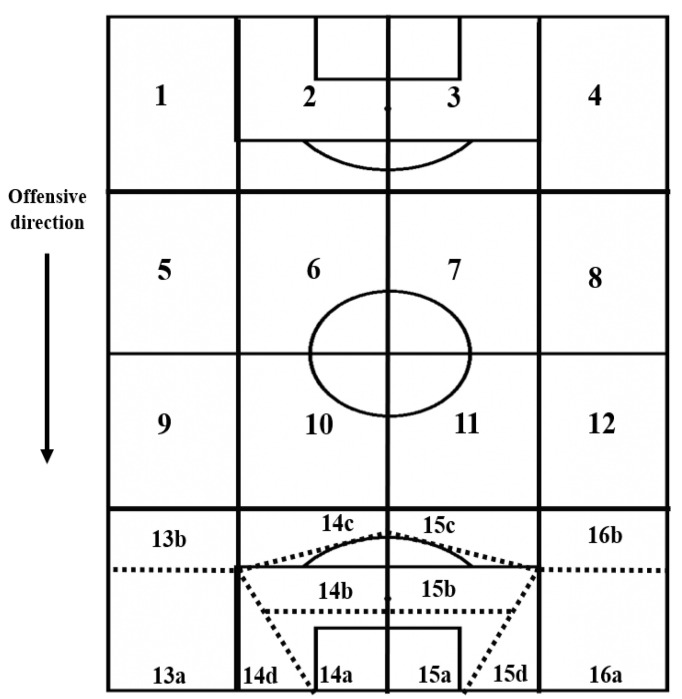
Zones of the field and “score pentagon”. The “score pentagon” is subdivided into several areas to perform a more detailed evaluation of goals and goal scoring opportunities.

When the player receives the ball, six different dimensions are analyzed to capture the spatial and tactical context where the player starts the IBP (Table 1). To capture the location of the players, two key dimensions were created. First, the field space was divided into four sectors and four channels, forming sixteen different zones that evaluate the specific location of the players in relation to the official soccer field (Figure 2). It is important to highlight that in the offensive sector, specific zones were created based on the scoring pentagon, which a determined space where there is less than 20 m from the goal and exists high shooting angle, aspects that are crucial to score goals [40,41,42]. According to this pentagon, different areas are subdivided into zones in order to perform a more specific analysis of the individual possessions that achieve goals or scoring chances [42].

**Figure 3:**
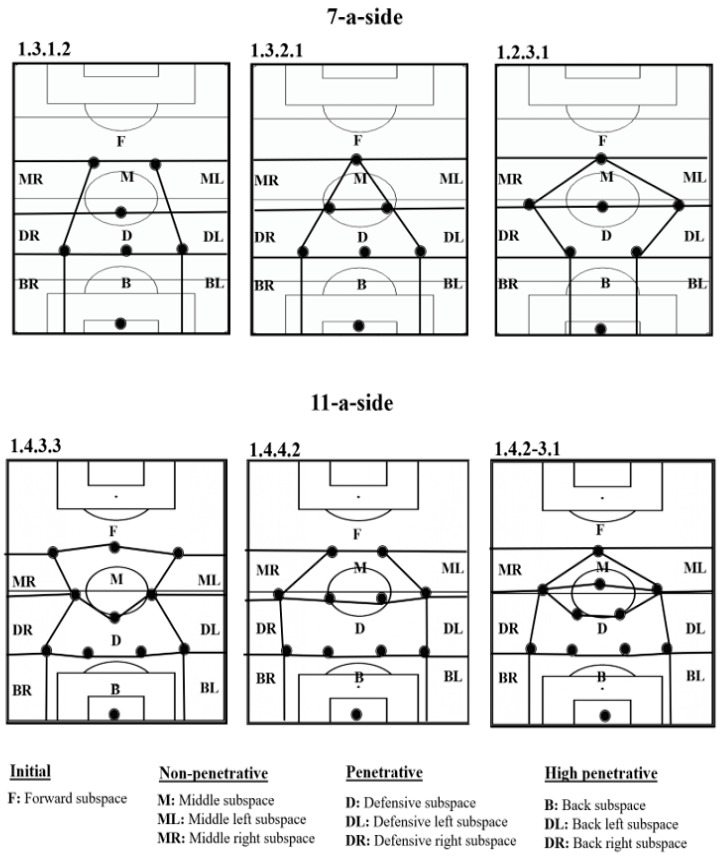
Different subspaces of the space of defensive occupation according to the tactical formation both in 7- and 11-a-side soccer.

Secondly, this tool evaluates the location of the player in relation to the position of the defensive team. This fact aims to evaluate the players´ actions in a representative spatial and tactical context. For this purpose, the opponent interaction is captured using the space of defensive occupation (SDO) [43] that in our tool is called “invasive space” because the location of the player within this space shows the level of invasion over the opposing defensive organization. The SDO was defined by Grehaigne [44] as the space that is formed by the positions of the players located, at a specific moment, in the periphery of a team in play, except the goalkeeper. This defensive shape creates different subspaces that are dynamic and change every second depending on the movement of the players. In this way, the location of the ball carrier in relation to the SDO of the opponent is evaluated based on ten subspaces that have been created considering previous studies [42,43,45]. It is crucial to mention that the different subspaces of the SDO must be adapted depending on the competition format (7-a-side vs. 11-a-side) and tactical formation. Figure 3 shows the different subspaces according to the most used defensive tactical formations both in 7-a-side and 11-a-side [46,47,48]. In addition to the invasive space, the “defensive pressure” is the other dimension related to opponent interaction that reflects the closeness and behavior of defensive players in relation to the attacker with the ball when receiving the ball possession.

Furthermore, the INDISOC considers the offensive tactical scenario where the player receives the ball (Table 1). For that purpose, the dimension “offensive support” and “type of attack” are analyzed. The first dimension shows the number of possible passing options that the observed player has in the moment of receiving the ball. This dimension reflects the influence of the team positioning and available passing lanes on individual performance. Moreover, evaluating the type of attack contributes to contextualize the collective organization and the moment of play when the IBP takes place [49].

**Table 1:** Operational definitions for the dimensions included in the moment of receiving the ball.

Dimension	Categories	Subcategories
**1. Field zone** Zone of the field where the player receives or recovers the ball (Figure 2)	**Defensive sector**	1; 2; 3; and 4.
	**Pre-defensive sector**	5; 6; 7; and 8
	**Pre-offensive sector**	9; 10; 11; and 12.
	**Offensive sector**	13a, 13b, 14a, 14b, 14c, 14d. 15a, 15b, 15c, 15d, 16a, 16b.
**2. Invasive space** Area within the SDO of the opponent where the player receives the ball (Figure 3)	**Initial subspace**	Forward zone **(F)**
	**Non-penetrative subspace**	Middle Right zone **(MR)**, Middle Left Zone **(ML)**, Middle zone **(M)**
	**Penetrative subspace**	Defensive right zone **(DR)**, defensive left zone **(DL)**, defensive zone **(D)**
	**High-penetrative subspace**	Back right zone **(BR)**, back left zone **(BL)**, back zone **(B)**.
**3. Defensive pressure** Distance between the player with the ball and the immediate pressuring of opponent player(s) during the first three seconds of ball possession	**Initial pressure:** one or several opponent players pressure the attacker within the first 3 s of the possession (the defender(s) are located within 1.5 m of the player) **Non-initial pressure:** any player pressures the attacker during the first 3 s of the possession.	
**4. Body shape** Body orientation with respect to the opponent goal at the moment of receiving the ball	**Facing the goal:** Player’s chest is facing the opposing goal **Facing right:** Player’s chest is facing the right line in relation to the opposing goal **Facing left:** Player’s chest is facing the left side in relation to the opposing goal **Back to goal:** Player’s back is facing the opposite side of the opposing goal.	
**5. Offensive support** Number of passing options that the on-the-ball attacker possesses at the moment of receiving the ball possession.	**Offensive support**	**Many options:** The player has open passing lanes with three or more teammates **Few options:** The player has open lanes with one or two teammates
	**No offensive support**	**No options:** The player has no open passing lanes with his/her teammates.
**6. Type of attack** Degree of offensive directness in the offensive process [14,50].	**Positional attack:** (a) The team possession begins by winning the ball in play or restarting the game, (b) the opposing team is prepared defensively, and (c) the circulation of the ball takes place more in width than in depth [50] and the intention of the team is to disorder the opponent using either fast, direct, or combinative play. **Counterattack:** (a) The team possession begins by winning the ball in play, (b) the progression towards the goal attempts to utilize a degree of imbalance from start to the end with high tempo [14], (c) the intention of the team is to exploit the space left by the opposing team when they were attacking, and (d) the opposing team does not have the opportunity to reorganize their system and be prepared defensively.	

### 2.5. Processing the Ball

Immediately after receiving the ball, the INDISOC analyzes the type of ball processing actions that players decide to perform by differentiating between quick actions based on receiving/passing and actions based on carrying the ball and dribbling (Table 2).

### 2.6. Culminating the Action

After processing the ball and depending on the tactical scenario, players can decide between multiple options to culminate their action and contribute to the success of the team’s offensive sequence (Table 3). This moment is evaluated by the INDISOC tool by analyzing five dimensions. The first dimension is the type of “action culmination”, which evaluates the degree of penetration over the opponent that players try to achieve with their actions based on the offensive principles of play. In addition to evaluate the tactical intention, this dimension includes subcategories that specify the technical–tactical actions executed by the players. For example, this dimension not only register if the player tries to shoot at goal, but also what type of shot performs, differentiating between volleys, headers, and one-touch shots, etc. Thus, this dimension offers a complete analysis of players’ tactical and technical performance when culminating their individual actions. To tactically contextualize this action, the field zone, and the invasive space of the opponent where it takes place, are also registered within this moment.

Finally, the tactical outcome of the IBP was evaluated by analyzing two dimensions. On one hand, the dimension “next player” registers the connection between the observed player and the next receiving player, when the culminating action is a pass. This evaluation allows evaluating the passing interaction between players and quantifying the most frequent passing networks of the observer player. On the other hand, the last dimension called “tactical outcome” is key to quantify the success of the action depending on the achieved performance. In this sense, the player will achieve a successful action if the pass connects to a receiving player, or the shot achieves goal. Additionally, an IBP is considered successful if the observed player receives a foul when processing the ball or achieves a corner-kick or throw-in for the attacking team. This last dimension can be used as a dependent variable in research studies to check the interactive effect of the previous independent dimensions on the tactical success of individual ball possessions.

In Table 4, a practical example of the analysis of a specific IBP in 11-a-side soccer can be observed. In this example, the three moments of the IBP are shown graphically, where the key task and individual constraints under analysis are highlighted, such as the invasive space, the field zone of intervention, the offensive support, body shape, and the action culmination. Additionally, in this specific example, the tool shows the requirement of completeness, so that any behavior under analysis could be assigned to one of the categories and subcategories. Additionally, the tool meets the criterion of mutual exclusivity, since there is not overlap of the categories and each analyzed behavior was assigned to a single category [38].

### 2.7. Statistical Analysis

To evaluate the inter- and intra-observer concordance of the INDISOC dimensions, Cohen’s kappa index [51] was calculated using the LINCE-PLUS software [52].

## 3. Results

Table 5 shows the values of content validity in form of Aiken’s V coefficients for each of the dimensions that form the INDISOC tool. It can be observed that all dimensions presented values higher than 0.85 both for pertinence and comprehension. Additionally, a lower average value was obtained in comprehension (0.93) than in pertinence (0.97).

Table 6 shows the Kappa (K) values for each of the dimensions of the INDISOC observational tool. In general, all K values were higher than 0.73 for the sub-categories section and 0.81 for the categories section.

The K values of the inter-observer analysis were lower (0.73–0.98) than the values of the intra-observer analysis (0.79–1). Furthermore, the K values for the categories were higher than when these categories were divided into sub-categories both for inter-observer (0.85–0.97 vs. 0.73 vs. 0.93) and intra-observer (0.89–1 vs. 0.79–0.98), respectively.

The lowest K values were obtained in dimensions related to the invasive space both for the moment of receiving the ball (0.73–0.89) and culminating the action (0.76–0.87). On the other hand, the highest K values were observed for dimensions related to the field zones (0.90–1), type of attack (0.95–1), and tactical outcome (0.85–0.98).

## 4. Discussion

The aim of this study was to design and check the reliability of an observational tool to analyze the individual offensive behavior in competitive 7- and 11-a-side soccer.

Firstly, this paper presented the design process of the INDISOC tool, which provides an observational framework to analyze individual offensive behavior in competitive soccer, and especially in youth players. In comparison with previous instruments, this tool not only evaluates the individual behavior, but also considers the influence of environmental and task constraints such as contextual variables, spatial location, defending positioning, and pressure, as well as the team’s collective organization and passing support. In fact, some of the dimensions included in this instrument such as the invasive space, defensive pressure and type of attack have been adapted from the REOFUT tool [42], which is designed to analyze the collective performance in soccer. The inclusion of real game constraints provides greater contextualization of the players’ behavior, as previous studies claimed [32,33,53], and gives researchers the possibility to analyze the interactive effects of multiple dimensions on individual behavior and performance.

Additionally, this observational tool is different from previous instruments such as the GPET [20], IMLPFoot [21], or FOCOS [23] because of its exclusive focus on analyzing the real 7- and 11-a-side competition, which can complement the information provided by the mentioned tools in training formats. In fact, the INDISOC shares with tools such as the GPAI [17], GPET [20], or FUT-SAT [19] the evaluation of individual behaviors considering their relationship with offensive principles of play [54]. However, one of the most crucial aspects of the INDISOC is to describe in detail the type of technical–tactical actions that can be performed both for processing the ball and culminating the actions, focusing on its tactical functionality rather than solely its execution, as some authors suggested [55]. In this sense, the INDISOC tool presents some similar features in relation to the observational framework created by Ortega-Toro et al. [22]. Specifically, both tools organize the observation in the same three temporal moments of the IBP (start, development, and end) and evaluate some common dimensions such as the player’s offensive support and type of technical actions performed. However, the INDISOC tool focuses its analysis on the implementation of the offensive principles of play, as well as evaluates the technical–tactical performance with different categories. In fact, the format of this tool that includes categories and subcategories allows researchers to have two levels of analysis to modulate the specificity of the tactical evaluation. Considering the subcategories, the INDISOC permits not only to evaluating the tactical principle implemented by the player (i.e., possess, progress, finish), but also analyzing in detail the type of action executed (i.e., penetrative pass, key pass, cross, header, volley, etc.).

Secondly, this paper checked the reliability of INDISOC through the analysis of agreement according to the proposal of the Kappa index, considered as a suitable method to measure the agreement for categorical data in sport performance [56]. In this sense, the INDISOC dimensions showed an excellent level of reliability according to the criteria of previous studies [57,58]. Specifically, the inter-observer analysis registered lower levels of reliability than the intra-observer one. This fact may be possible due to the nature of some dimensions, where the interpretation by the observers has a greater importance and, therefore, some observations may naturally be more complex to perform more accurately than others [59].

For instance, the dimensions “field zone”, “body shape”, “type of attack”, “next teammate”, and “field zone of culmination” registered K values higher than 0.90 both for categories and subcategories. This high level of reliability is probably due to a lower degree of interpretation by the observers when analyzing the game actions. However, the two dimensions related to the invasive space obtained the lowest levels of agreement, especially considering the analysis of subcategories (inter-observer = 0.73–0.86; intra-observers: 0.79–0.89). This fact could be related to the dynamic nature of the subspaces within the SDO that change every second during the IBP and require a higher degree of interpretation by the observers. Nevertheless, our analysis agrees with previous studies in demonstrating that the evaluation of the SDO can be a reliable measure for the analysis of the game space in soccer [42,43,45].

The presented tool has several limitations. On one hand, the tactical evaluation is only focused on the offensive moment and, particularly, on the attacker with the ball. Thus, this tool does not analyze the offensive behaviors off the ball and the defensive moment. This fact, despite being reductionist considering the globality of the game, makes this tool more specific and focuses exclusively on the actions with the ball, which have a key weight in the offensive individual and collective performance. On the other hand, this tool is based on notational analysis and therefore on the observation, interpretation, and recording of events that occur during the game. This method may not entirely capture the complex and interactive nature of individual tactical actions during the game, as some authors have claimed [60,61,62]. Finally, the next limitation can also be considered an opportunity for development in the future. INDISOC has been designed and validated only for the analysis of 7- and 11-a-side competitive soccer. Thus, the next step for this research group should be to explore its adaptation to analyze small-sided games, which would expand its use in multiple contexts.

Nevertheless, the INDISOC provides relevant applications both for the academic and professional fields. Academically, this tool can be used for researchers to perform case-studies of players, playing positions, specific teams, or competitions in both 7-a-side and 11-a-side soccer. In this vein, the design of this tool allows researchers to carry out descriptive, comparative, and predictive analyses, where the combined and interactive effects of environmental, task, and individual constraints on tactical performance can be evaluated. Additionally, this tool can be used in conjunction with other types of data (positional data) in order to create mixed-method (quantitative and qualitative) analyses. From a professional perspective, this tool can be useful for coaches and performance analysts especially in youth soccer to evaluate the individual performance of specific players and design appropriate learning processes in children and adolescent players.

In conclusion, the results of the present study indicated optimal inter- and intra-reliability levels, suggesting that the INDISOC observational tool could be a suitable tool for analyzing individual offensive behavior in competitive 7- and 11-a-side soccer.

## Figures and Tables

**Table 2:** Operational definitions for the dimensions included in the moment of processing the ball.

Dimension	Categories	Subcategories
**7. Type of action.** Behavior of the ball carrier since he/she receives the ball until the culmination of his/her action.	**Receiving and passing**	**One-touch action:** The ball carrier only needs one contact with the ball to culminate his/her action. **Quick action (2–4 ball touches):** the ball carrier needs few contacts with the ball to culminate his/her action
	**Running with the ball**	**Carrying the ball:** the ball carrier runs with the ball performing multiple touches or directional changes. **Dribbling:** The ball carrier attempts to beat an opponent in possession of the ball

**Table 3:** Operational definitions for the dimensions included in the moment of culminating the action.

Dimension	Categories	Subcategories
**8. Action culmination:** Final action of the player that intends to pass to a teammate or to shoot at the goal	**Possess**	**Non-penetrative pass:** the ball carrier performs a pass that does not past opponent player(s)
	**Progress**	**Penetrative pass:** the ball carrier performs a pass towards the opponent’s goal past opponent player(s) Key pass: the ball carrier performs a pass from central channels of the field that breaks the opposing defensive line.
	**Assist**	**Key pass with assist:** the ball carrier performs a pass from central channels of the field that breaks the opposing defensive line and allows the receiver to have an immediate scoring opportunity **Cross:** the ball carrier performs a pass from the exterior channels of the field in the opposing half (Figure 2) towards the penalty box **Goal pass:** the ball carrier performs a pass from the penalty box that allows the receiver to have an immediate scoring opportunity
	**Finish**	**Shot-feet:** the ball carrier shoots at the goal while the ball is on the ground using two or more contacts to the ball. **Shot-feet (one-touch):** the ball carrier shoots at the goal while the ball is on the ground using one single contact to the ball **Header:** the ball carrier shoots at the goal while the ball is in the air by heading the ball. **Volley:** the ball carrier shoots at goal while the ball is in the air by using one single contact to the ball
	**Other**	**No culmination:** The ball carrier does not achieve ball possession. **Long ball:** The ball carrier performs a long distance (+40 m) and high pass without a clear receiver. **Clearance:** The ball carrier clears the ball away without a defined offensive purpose.
**9. Field zone of culmination** Zone of the field where the player performs the final action of the IBP (Figure 2)	**Defensive sector**	1; 2; 3; and 4.
	**Pre-defensive sector**	5; 6; 7; and 8
	**Pre-offensive sector**	9; 10; 11; and 12.
	**Offensive sector**	13a; 13b; 14a; 14b; 14c; and 14d. 15a; 15b; 15c; 15d; 16a; and 16b.
**10. Invasive space of culmination** Area within the Space of Defensive Occupation (SDO) of the opponent where the observed player performs the final action of the IBP (Figure 3)	**Initial subspace**	Forward zone **(F)**:
	**Non-penetrative subspace**	Middle Right zone **(MR)**, Middle Left Zone **(ML)**, Middle zone **(M)**
	**Penetrative subspace**	Defensive right zone **(DR)**, defensive left zone **(DL)**, defensive zone **(D)**
	**High penetrative subspace**	Back right zone **(BR)**, back left zone **(BL)**, back zone **(B)**.
**11. Individual tactical outcome:** Final performance of the action, considering the success when passing/shooting.	**Successful (1)**	Pass completed/Goal/Foul received, corner or throw-in achieved.
	**No Successful (0)**	Pass intercepted/missed/Shot off target./Ball out of play/Ball lost by tackle/Turnover/Foul committed/No control of the ball:.
12. Next teammate *: Receiving player when the observed player performs a pass.	**Goalkeeper**	Goalkeeper
	**Full back**	Left full back; Right full back
	**Central back**	Right Central Back; Left Central Back
	**Midfielders**	Defensive midfielder; Offensive midfielder
	**Wingers**	Right winger; Left winger
	**Forwards**	Forward
	**No connection**	The observed player does not perform a pass

* The categories and sub-categories of this dimension may be adjusted depending on the team formation used by the observed team.

**Table 4:** Example of analysis of the IBP using the dimensions of the INDISOC tool.

Moment	Dimension	Category	Subcategory
**Receiving the ball**			
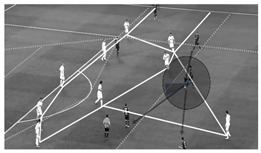	**1. Field zone** **2. Invasive space** **3. Defensive pressure** **4. Body shape** **5. Offensive support** **6. Type of attack**	Pre-offensive Non-penetrative No pressure Facing forward Offensive support Organized attack	10 Middle zone - - Many options -
**Processing the ball**			
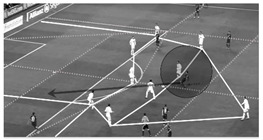	**7. Type of action**	Controlling and passing	Quick action
**Culminating the action**			
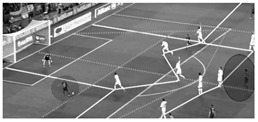	**8. Action culmination** **9. Field zone of culmination** **10. Invasive space of culmination** **11. Next player** **12. Tactical outcome**	Assist Offensive Non-penetrative Wingers Positive	Key pass with assist 14c Middle zone Left winger Pass completed

**Table 5:** Values of content validity (Aikens’s V) of the dimensions that form the INDISOC observational tool.

Moment	Dimension	Aikens’s V	
		Pertinence	Comprehension
**Receiving the ball**	1. Field zone	1	0.92
	2. Invasive space	0.95	0.91
	3. Defensive pressure	1	0.87
	4. Body shape	1	0.97
	5. Offensive support	1	0.95
	6. Type of attack	0.95	0.88
**Processing the ball**	7. Type of action	1	0.92
**Culminating the action**	8. Action culmination	0.95	0.95
	9. Field zone of culmination	1	0.92
	10. Invasive space of culmination	0.95	0.91
	11. Next teammate	0.85	0.87
	12. Tactical outcome	1	0.87
**Average score**		0.97	0.93

**Table 6:** Kappa values obtained for the dimensions of the INDISOC observational tool in 7-a-side and 11-a-side soccer.

7-a-Side Soccer					
Moment	Dimension	*K* Inter-Observer		*K* Intra-Observer	
		Categories	Subcategories	Categories	Subcategories
**Receiving the ball**	1. Field zone	0.93	0.90	0.98	0.93
	2. Invasive space	0.83	0.73	0.93	0.86
	3. Defensive pressure	0.82	-	0.95	
	4. Body shape	0.95	-	0.98	
	5. Offensive support	0.90	0.84	0.91	0.88
	6. Type of attack	0.95	-	0.98	
**Processing the ball**	7. Type of action	0.91	0.82	0.97	0.95
**Culminating the action**	8. Action culmination	0.89	0.82	0.99	0.93
	9. Field zone of culmination	0.94	0.90	1	0.98
	10. Invasive space of culmination	0.81	0.74	0.88	0.84
	11. Next teammate	0.95	-	1	
	12. Tactical outcome	0.92	0.85	0.96	0.91
**11-a-Side Soccer**					
**Receiving the ball**	1. Field zone	0.97	0.92	1	0.96
	2. Invasive space	0.86	0.78	0.89	0.81
	3. Defensive pressure	0.88	-	0.94	
	4. Body shape	0.93	-	0.98	
	5. Offensive support	0.93	0.85	0.97	0.92
	6. Type of attack	0.97	-	1	
**Processing the ball**	7. Type of action	0.98	0.87	1	0.97
**Culminating the action**	8. Action culmination	0.91	0.81	0.95	0.89
	9. Field zone of culmination	0.98	0.93	1	0.98
	10. Invasive space of culmination	0.85	0.76	0.87	0.79
	11. Next teammate	0.97	-	1	
	12. Tactical outcome	0.94	0.91	0.98	0.96

## Data Availability

The data that support the findings of this study are available from the corresponding author, [R.A.], upon reasonable request.
